# Investigating the gaze‐driven reversed congruency effect in the spatial Stroop task: A distributional approach

**DOI:** 10.1111/bjop.70004

**Published:** 2025-06-20

**Authors:** Renato Ponce, Juan Lupiáñez, Carlos González‐García, Maria Casagrande, Andrea Marotta

**Affiliations:** ^1^ Department of Psychology Sapienza University of Rome Rome Italy; ^2^ Department of Experimental Psychology & Mind, Brain and Behaviour Research Centre (CIMCYC) University of Granada Granada Spain; ^3^ Department of Dynamic and Clinical Psychology, and Health Studies Sapienza University of Rome Rome Italy

**Keywords:** delta plots, distributional analysis, gaze and arrows, social attention, spatial Stroop

## Abstract

This study investigated how social (faces and eyes) and non‐social (arrows) stimuli modulate attentional orienting in a spatial Stroop task, using a distributional approach. Data from 11 studies (*N* = 705) were analysed through cumulative distribution functions (CDF), delta functions, and polynomial trend analyses. Three models were applied: (1) a reaction time (RT) model comparing social (faces and eyes) vs. non‐social stimuli under congruent and incongruent conditions, (2) a delta model assessing conflict effects across quantiles, and (3) a trend model identifying specific delta function patterns. Non‐social targets produced a standard congruency effect (SCE), with faster responses in congruent trials but no consistent conflict reduction across the distribution. In contrast, social stimuli exhibited a reversed congruency effect (RCE), with faster responses in incongruent trials, emerging from the second quantile onward and minimal conflict among the fastest responses. Social targets exhibited comparable reaction times in the RT model and similar early delta plot patterns, suggesting shared initial perceptual and attentional mechanisms between faces and eyes. However, faces eventually induced a larger RCE, possibly due to their more complex configuration. These findings highlight distinctive patterns between social and non‐social processing in the spatial Stroop task.

## BACKGROUND

The ongoing debate in social attention research focuses on whether social orienting – paying attention to where others are looking – is driven by general attentional mechanisms related to directional information, like eye‐gaze (Cole & Millett, 2019), or by specific social processes involving the interpretation of intentions and mental states (Apperly & Butterfill, 2009). To explore this, and given that arrows and gaze seem to orient attention similarly in standard arrow/gaze cueing paradigms (Chacon‐Candia et al., 2023), recent studies have used spatial interference paradigms, such as the spatial Stoop (Cañadas & Lupiáñez, 2012; Jones, 2015; Marotta et al., 2018; Narganes‐Pineda et al., 2022), to investigate whether social stimuli uniquely activate attentional processes.

In these tasks, stimuli like arrows (non‐social) or eyes (social) appear on either side of a central fixation, pointing or looking left or right. Participants respond to the direction of the stimuli while ignoring their spatial location, creating congruent trials (e.g. arrows/gaze directed right on the right side) and incongruent trials (e.g., arrows/gaze directed right on the left side). Arrows typically produce a standard congruency effect (SCE), with faster responses in congruent trials. In contrast, gaze stimuli produce an opposite effect, known as the reversed congruency effect (RCE), where responses are faster in incongruent trials. This phenomenon has been observed in several studies (Cañadas & Lupiáñez, 2012; Dalmaso et al., 2023; Jones, 2015; Marotta et al., 2018; Narganes‐Pineda et al., 2022), suggesting that gaze triggers specific attentional processes distinct from those activated by other types of stimuli.

The SCE is generally attributed to the asymmetric relationship between stimulus dimensions –specifically, the task‐relevant direction and the task‐irrelevant spatial location relative to lateralized motor responses (Lu & Proctor, 1995; Viviani, Visalli, Montefinese, et al., 2024). Despite instructions to focus on direction, irrelevant location information consistently influences responses, slowing reaction times (RTs) in incongruent trials. According to Kornblum's (1992) taxonomy, this setup forms a Type 8 ensemble, characterized by three dimensions overlap: between relevant and irrelevant stimulus dimensions, relevant stimulus and response dimensions, and irrelevant stimulus and response dimensions. This requires participants to resolve conflict at various levels – task, stimulus, and response – affecting the overall performance (Banich, 2019; De Houwer, 2003; Parris et al., 2022; Viviani, Visalli, Montefinese, et al., 2024).

However, the mechanisms driving the interaction between task‐relevant and irrelevant information with gaze stimuli, contributing to the RCE, remain under discussion. The reversed effect has led to multiple theories, categorized into social (Cañadas & Lupiáñez, 2012; Edwards et al., 2020; Hemmerich et al., 2022; Marotta et al., 2018) and non‐social (Chen et al., 2022; Tanaka et al., 2024, 2025) explanations.

Social theories suggest that gaze processing triggers specific mechanisms that facilitate the RCE. The eye‐contact hypothesis (Cañadas & Lupiáñez, 2012; Marotta et al., 2018) proposed that incongruent gaze trials might be implicitly perceived as looking at the participant, leading to faster responses. This is supported by findings indicating that humans have a tendency to interpret gaze as direct when faced with perceptual uncertainty (Mareschal et al., 2013). However, Narganes‐Pineda et al. (2022) challenged this, showing that explicit attention to gaze direction is required for the RCE. In their study, when participants had to discriminate the colour of the eyes rather than the direction of gaze, so that task‐relevant information changed from direction to colour (i.e., changing the overlapping dimensions), the RCE was not observed.

Edwards et al. (2020) proposed an alternative explanation, suggesting that the RCE might result from a joint attention episode, where incongruent eyes appear to ‘look’ at the fixation point where participants are focused. However, the reversion was absent in children under 12 (Aranda‐Martín et al., 2022), despite joint attention developing by age 4 (Mundy et al., 2007). Hemmerich et al. (2022) later proposed that congruent trials might shift attention away from the task, slowing response times in this condition, thus leading to the RCE. Yet, Aranda‐Martín et al. (2023) found no effect on the RCE when using objects or frames to prevent distraction, challenging this hypothesis.

More recently, an integrated framework has been developed that combines general attentional processes with specific social mechanisms, interacting with perceptual features and task dynamics to facilitate or enhance the RCE (Hemmerich et al., 2022; Marotta et al., 2019; Ponce et al., 2024; Román‐Caballero et al., 2021a, 2021b). In this view, general attentional processes related to the direction and spatial location of stimuli create similar interference for both arrows and gaze, leading to faster responses in congruent conditions. However, gaze stimuli activate additional processes, incorporating new information that can reverse the typical interference effect, resulting in the RCE.

More specifically, the spatial interference may arise from the interaction of three vectors (Hemmerich et al., 2022): (a) the relevant stimulus direction, (b) its irrelevant spatial location – both common to social and non‐social targets – and (c) a ‘looking’ vector, unique to social stimuli. The interaction between the relevant and irrelevant dimensions would lead to standard congruency for both social and non‐social targets. However, the third vector conveys socially meaningful information (Hietanen, 2018), and may be strong enough to override the SCE observed for gaze targets, thereby contributing to the emergence of the RCE (Hemmerich et al., 2022; Ponce et al., 2024; Román‐Caballero et al., 2021a, 2021b). Indeed, gaze has been shown to play a fundamental role in multiple stages of social cognitive processing (Brignani et al., 2009; Mundy, 2018; Yang et al., 2015), facilitating mechanisms related to gaze perception (Kobayashi & Kohshima, 1997), shifts in gaze direction (Gobbini & Haxby, 2007), gaze following (Emery, 2000), and theory‐of‐mind‐related processes (Baron‐Cohen, 1994). These processes may account for the strength of the looking vector in the direction where the stimulus is ‘looking’, which can lead to either slower responses in congruent trials or faster responses in incongruent ones.

This framework is supported by electrophysiological, neuroimaging, and behavioural data. Marotta et al. (2019) found that early event‐related components (P1, N1) were similarly affected by congruency in both gaze and arrow stimuli, while later components (N2 and P3) showed opposite patterns. Moreover, although the N170 was not modulated by congruency, differences between arrows and eyes were noted, possibly linked to their nature (Bentin et al., 1996; Nemrodov et al., 2014). An fMRI study by Narganes‐Pineda et al. (2023) revealed similar activations in right parieto‐temporo‐occipital regions during conflict resolution for both gaze and arrow stimuli, along with distinct activations in the frontal eye field (FEF) and occipital regions.

Behaviourally, Román‐Caballero et al. (2021a, 2021b) found that factors reducing the SCE with arrows, such as increased perceptual background complexity,^1^ which hindered the extraction of task‐relevant information, simultaneously amplified the RCE in gaze trials. The need for background segregation before processing directional information – like extracting gaze direction from eyes within a face – created a temporal gap between relevant and irrelevant information. This gap allowed irrelevant information to decay (*temporal‐delay* hypothesis; Hommel, 1993), reducing spatial conflict and enhancing the RCE for gaze targets. In addition, a developmental study showed that while a typical SCE was present in children as young as 4 years old and continued through childhood for both gaze and arrows, the RCE specifically observed for gaze stimuli only emerged around age 12 (Aranda‐Martín et al., 2022).

Further evidence supports the social interpretation of gaze in producing the reversion. The RCE appears to be influenced by the emotional facial expressions (Jones, 2015; Marotta et al., 2022; Torres‐Marín et al., 2017), modulated by social familiarity (Ishikawa et al., 2024), and negatively correlated with social anxiety scores (Ishikawa et al., 2021). While the RCE has been compared with other social cues like pointing hands, the reversion was only observed with gaze targets (Bonventre & Marotta, 2023; Dalmaso et al., 2023). The RCE has also been noted with certain animal faces (Ishikawa et al., 2024), inverted faces (Tanaka et al., 2023), and using oral responses (Narganes‐Pineda et al., 2022, experiment 3A).

Alternatively, non‐social explanations for the RCE focus on perceptual factors (Chen et al., 2022) or task‐related effects (Tanaka et al., 2024, 2025). Chen et al. (2022), for instance, found that stimuli with similar perceptual features, whether social or non‐social, produced the SCE (although see Cañadas & Lupiáñez, 2012), suggesting that perceptibility influences congruency effects. They also suggested that variations in other studies might result from changes in attentional focus, as described by the zoom lens model (Eriksen & St. James, 1986).

Tanaka et al. (2024, 2025) introduced the dual‐stage hypothesis, challenging the idea that the RCE is exclusively related to gaze mechanisms. After observing the RCE with tongues as directional targets, they built on the findings of Román‐Caballero et al. (2021a, 2021b), and the *activation–suppression* hypothesis (Ridderinkhof, 2002a, 2002b) an account that focuses on the temporal dynamics. In the first stage, segregating a target from a complex background (i.e., extracting directional information from eyes within a face) delays the extraction of directional information, allowing the location code to decay (Hommel, 1993). In the second stage, selective attention suppresses irrelevant spatial codes, inhibiting conflict, which ultimately leads to the RCE. Specifically, in congruent conditions – where spatial and directional codes align (e.g., a right‐looking gaze in the right visual field) – selective inhibition may suppress the correct spatial code (right), delaying the response. Conversely, in incongruent conditions – where the spatial and directional codes do not align (e.g., a right‐directed gaze in the left visual field) – selective inhibition suppresses the incorrect spatial code (left), speeding up the correct responses (right). For simpler perceptual stimuli like arrows, however, responses occur before response inhibition sets in, making congruent responses faster.

Overall, the RCE appears to arise from a complex interplay between social and non‐social components, including perceptual features and temporal‐related effects, that modulate processing at multiple levels. This view is consistent with the dimensional overlap model (Kornblum, 1992, 1994) and the multiple stage/loci theories of Stroop interference (Banich, 2019; De Houwer, 2003; Parris et al., 2022), providing a useful framework for assessing these patterns (Viviani, Visalli, Montefinese, et al., 2024). By integrating this framework and building on previous research emphasizing temporal dynamics (Hemmerich et al., 2022; Marotta et al., 2019; Ponce et al., 2024; Román‐Caballero et al., 2021a, 2021b; Tanaka et al., 2025), the current exploratory study employs a distributional analysis to deepen our understanding of how social and non‐social stimuli affect attentional mechanisms.

In conflict tasks, a distributional approach provides tools to examine how congruency effects evolve over time (Balota & Yap, 2011; Mittelstädt & Miller, 2020; Smith & Ulrich, 2025; Speckman et al., 2008; Torres‐Quesada et al., 2022). By segmenting response latency distributions into fastest, intermediate, and slowest responses, we gain clearer insights into underlying mechanisms. This method aligns with the dual‐route models, which propose that task‐relevant information is processed by controlled mechanisms, while task‐irrelevant information is handled by automatic, transient processes (Banich, 2019; Cohen et al., 1990; De Jong et al., 1994; Ridderinkhof, 2002a, 2002b; Ulrich et al., 2015; see also direct and indirect route, for example, Luo & Proctor, 2019).

Most distributional research on spatial interference has centred on the Simon and Simon‐like tasks, establishing robust predictions and analytical frameworks (Burle et al., 2014; De Jong et al., 1994; Miller & Schwarz, 2021; Mittelstädt & Miller, 2020; Pratte et al., 2010; Proctor et al., 2011; Smith & Ulrich, 2025; Ulrich et al., 2015). Given that the spatial Stroop shares key similarities with the Simon task (i.e., irrelevant location‐based activation), these same distributional tools offer valuable insights for spatial Stroop research, applied within the framework of social attention.

Thus, the aim of the current study is to investigate the differences in attentional mechanisms involved in resolving spatial conflicts when using social stimuli (gaze) versus non‐social stimuli (arrows) in spatial Stroop tasks. Employing a distributional approach, we analyse how congruency effects vary in response latencies, providing deeper insights into the temporal dynamics underlying social and non‐social attentional orienting. Additionally, we examine whether the perceptual complexity of the background – such as the difference between full faces and isolated eyes – affects the pattern of the RCE in gaze stimuli.

Previous findings indicate that embedding stimuli within a complex background can modulate spatial interference effects, likely by increasing perceptual demands and hindering the extraction of task‐relevant information (Román‐Caballero et al., 2021a, 2021b; Tanaka et al., 2024, 2025). This interpretation is consistent with findings from location‐based conflict tasks, where interference is modulated by factors, such as reduced visibility (Mittelstädt & Miller, 2020), added conflicting layers (Scerrati et al., 2017), or decreased target discriminability (Ellinghaus et al., 2024). Within this framework, full‐face stimuli – compared with isolated eyes – are thought to introduce greater visual complexity by embedding directional cues within a richer social and perceptual context (Ponce et al., 2024; Román‐Caballero et al., 2021a and Tanaka et al., 2024). To investigate how these differences shape the time course of conflict resolution, we employed both the cumulative distribution function (CDF) and the delta function.

The CDF enables exploration of RT behaviour for congruent and incongruent trials at different distribution points (Ambrosi et al., 2019; Leber et al., 2013; Ulrich et al., 2015), allowing comparisons between social and non‐social targets, as well as between faces and eyes (within social targets). Typically, when analysing mean RTs, social targets elicit slower RTs than non‐social targets (Bonventre & Marotta, 2023; Dalmaso et al., 2023; Ishikawa et al., 2021). Moreover, based on prior research suggesting that faces require greater target–background segregation demands (Ponce et al., 2024; Román‐Caballero et al., 2021a, 2021b; Tanaka et al., 2024, 2025), we expect faces to elicit slower RTs than isolated eyes, reflecting the increased complexity involved in extracting directional information.

In addition, the delta function – derived from the CDF – can reveal how conflict effects vary across the RT distribution (De Jong et al., 1994; Pratte et al., 2010; Schwarz & Miller, 2012; Smith & Ulrich, 2025; Speckman et al., 2008). Delta functions represent quantile differences in RT distributions between congruent and incongruent conditions, often visualized in delta plots. These plots can be further analysed using slope or polynomial contrast analyses to assess their shape (Burle et al., 2014; Debey et al., 2015; Pratte, 2021). Typically, a less positive slope indicates more efficient inhibitory control – whether through stronger suppression of irrelevant information or additional resource allocation for target processing (Hübner et al., 2010; Mittelstädt et al., 2023; Ridderinkhof, 2002a, 2002b; van Campen et al., 2014). However, and not mutually exclusive, other research suggests that the slope of the delta plots may also reflect the relative speed of processing irrelevant versus relevant information (Mackenzie et al., 2022; Miller & Schwarz, 2021). Based on Tanaka et al. (2025), we expect negative‐going delta plots, reflecting across quantiles a reduction of the SCE for non‐social targets and a larger RCE for social targets. Furthermore, if background segregation is essential for the reversion (as posited in the first stage of the dual‐stage hypothesis), it is crucial to examine whether faces and eyes diverge in the fastest responses, before the reversed effect becomes more pronounced.

Ultimately, by examining RT distributions through both the CDF and delta function, this study provides a general perspective on how social and non‐social stimuli impact attentional processes across different stages of cognitive processing, considering their underlying temporal dynamics.

## METHOD

### Studies

The studies included in this analysis are summarized in Table 1, covering 16 experiments and 705 participants. To ensure comparability, we applied the same selection criteria as Ponce et al. (2024): (1) use of the spatial Stroop task, (2) comparison of cropped‐eyes or faces as social targets with arrows as non‐social targets, (3) use of upright faces, and (4) implementation of an explicit discrimination task. Consequently, we excluded hand‐pointing targets (Bonventre & Marotta, 2023; Dalmaso et al., 2023), animal faces (Ishikawa et al., 2024), and inverted faces (Marotta & Lupiáñez, 2018; Tanaka et al., 2023). From Narganes‐Pineda et al. (2022), only data from the explicit task with motor responses (experiment 1) were included. Additionally, we did not consider the emotional expression condition (Marotta et al., 2022; Torres‐Marín et al., 2017), collapsing the data across target type.

**TABLE 1:** Studies selected for combined analyses.

N	Study	Sample	Target types	Observations
1	Torres‐Marín et al. (2017), experiments 1 and 2	40, 40	Face expressions	Data were collapsed across target types
2	Marotta et al. (2018)	35	Arrows vs. Eyes	
3	Marotta and Lupiáñez (2018)	17	Faces: Upright vs. Inverted	Inverted faces were removed
4	Marotta et al. (2019)	27	Arrows vs. Eyes	
5	Hemmerich et al. (2022), experiments 1 and 2	35, 33	Arrows vs. Eyes	
6	Narganes‐Pineda et al. (2022), experiment 1	24	Arrows vs. Eyes	
7	Marotta et al. (2022)	36	Face expressions	Data were collapsed across target types
8	Bonventre and Marotta (2023), experiments 1 and 2	24, 22	Arrows vs. Hand vs. Eyes	Hand targets were removed
9	Ishikawa et al. (2024), experiments 1 and 3	70, 54	Arrows vs. Cat Face/Robot Face vs. Eyes	Cat face and Robot face targets were removed
10	Tanaka et al. (2023), experiments 1A and 1B	38, 19	Faces: Upright vs. Inverted	Inverted faces were removed
11	Dalmaso et al. (2023)	191	Arrows vs. Hand vs. Face	Hand targets were removed

*Note*: The samples are separated by a comma when the study has more than one experiment.

Although some parameters varied across experiments, all the included studies shared the same ensemble type (Type 8) according to Kornblum's dimensional overlap taxonomy (Kornblum, 1992, 1994). In each task, participants were required to discriminate the direction of lateralized targets (faces, eyes, or arrows) while ignoring their spatial location, using left/right motor responses. This structural similarity aligns with the dual‐route models used in our interpretation of the results. To account for residual methodological heterogeneity, we used mixed‐effects models with a nested random structure that treated participants as nested within studies and modelled target type as study‐specific. This approach allows study‐level variability to be controlled statistically while preserving comparability in the aggregated analysis.

### Analyses and design

The analyses were conducted using RStudio (Posit team, 2024), with the script and datasets available on the OSF webpage (see data availability statement). To ensure consistency and comparability, we used the same cut‐off criteria for outliers and data pruning as in most original studies, excluding RTs shorter than 200 ms and longer than 1300 ms. We also repeated the analyses with non‐trimmed dataset (i.e., excluding only trials without responses), which revealed that the overall patterns remained stable for social targets (see Appendix S1). One participant from Dalmaso et al. (2023) was excluded due to a high rate of non‐responses (≈64%). Only correct responses were analysed, with the proportion of removed trials detailed in (Table S1).

The distributional analyses utilized the CDF and the delta function, employing the quantile‐averaging technique (De Jong et al., 1994; Ratcliff, 1979). The R quantile function was used (Grange, 2016; Mackenzie & Dudschig, 2021) to obtain five quantiles estimates at .1, .3, .5, .7, and .9 for each participant and experimental condition. Including five quantiles increases sensitivity to potential changes across the distribution, with the .5 quantile corresponding to the median (Grange, 2016; Hübner & Töbel, 2019; Smith et al., 2004). We applied the Type 8 quantile estimator as recommended by Hyndman and Fan (1996) to generate the CDF dataset for fitting the RT model. The delta function was derived by subtracting congruent quantiles from their corresponding incongruent ones, creating the delta dataset for fitting the delta model. The delta plot (Figure 2) was constructed by plotting delta function estimates against RT model estimates by quantile and target type.

Both RT and delta models were used to compare faces, eyes, and arrows, enabling us to examine distinctions between social and non‐social stimuli and to explore differences between social targets. The RT model compared response latencies based on congruency across the distribution, using a three‐way design: target type (face vs. eyes vs. arrows), congruency (congruent vs. incongruent), and quantile (five levels). The delta model analysed conflict effects across the distribution with a two‐way design: target type (face vs. eyes vs. arrows) and quantile (five levels). To further explore the consistency of the delta function patterns, analyses were repeated with four and 10 data points (quartile and decile models), with results detailed in Appendix S1. Additionally, to examine the trend of the delta plot, we employed an orthogonal polynomial contrast (Grant, 1956) by fitting the Trend model to the delta dataset – a method shown to better quantify the shape of the delta plot (see Burle et al., 2014; Pratte, 2021).

A linear mixed modelling (LMM) approach was applied using the lme4 (Bates et al., 2015) and lmerTest (Kuznetsova et al., 2017) R packages for model fitting and testing. The best‐fitting RT and delta models were identified through a stepwise procedure, which involved backward elimination of non‐significant effects (see Table S2). The RT model included target type, congruency, quantile (QQ), and their interactions as fixed effects. The delta model included target type, quantile, and their interaction. The Trend model used target type and mean RTs as predictors. Random effect structures accounted for study‐specific characteristics and complex nested data using crossed effects syntax (Schielzeth & Nakagawa, 2013). Participants were modelled as nested within studies, with target type treated as a study‐specific feature, considering also the distributional characteristics of each study as in Ponce et al. (2024). The rationale for these grouping structures is provided in Appendix S1.

Contrast analyses were performed using Tukey's HSD test within the mixed‐effects framework via the emmeans package in R (Lenth, 2023) for pairwise comparisons. One‐sample comparisons against zero were also conducted on delta estimated values to determine whether a conflict effect was elicited at specific data points using Bonferroni's adjustment. Reported values represent estimated marginal means for reaction times (EMM‐RT) and delta values (EMM‐∆) from the RT and delta models, respectively. Polynomial contrast analysis, comparing linear and quadratic coefficients, was performed using the emtrends function in emmeans, also with Bonferroni's adjustment. All contrast analyses employed 95% confidence intervals (CIs) and the Kenward–Roger method (Kenward & Roger, 1997) for estimating the degrees of freedom. Partial eta squared (η2p) was estimated using the effectsize R package (Ben‐Shachar et al., 2020), and effect sizes (d) for post‐hoc contrasts were calculated following Westfall et al. (2014; see also Brysbaert & Stevens, 2018), using the EMM‐RT and EMM‐∆ values.

## RESULTS AND DISCUSSION

The fitted models are summarized in Table 2. Stepwise analysis of the RT model revealed significant two‐way interactions between target type and congruency, target type and quantile, and congruency and quantile, leading to contrast analyses for these specific interactions. The delta model showed a significant interaction between target type and quantile. Unlike the other models, the Trend model, which was used for polynomial contrast to analyse the trend from the delta dataset, did not undergo stepwise analysis.

**TABLE 2:** Fitted models employed within the analyses.

Model	Formula
RT	RT ~ TargetType * Congruency + TargetType * QQ + Congruency * QQ + (1|Study:id) + (1|Study:TargetType) + (1|Study:TargetType:Congruency:QQ)
Delta	delta ~ TargetType * QQ + (1|Study:id) + (1|Study:TargetType) + (1|Study:TargetType:QQ)
Trend	delta ~ TargetType * poly(meanRT, degree = 2) + (1|Study:id) + (1|Study:TargetType) + (1|Study:TargetType:meanRT)

### RT model

The analysis of variance (ANOVA) on RT model showed a main effect of target type, *F*(2, 20.59) = 31.92, *p* < .0001, η2p = .756, with participants responding faster to arrows (508 ms, SE = 10.8) than to faces (610 ms, SE = 12.9) and eyes (615 ms, SE = 11.4), and no significant difference between face and eye targets. The main effect of congruency, *F*(1, 232.91) = 16.44, *p* < .0001, η2p = .066, showed faster RTs in incongruent trials compared with congruent ones. The quantile main effect, *F*(4, 232.91) = 2665.18, *p* < .0001, η2p = .979, indicated a consistent increase in RTs across quantiles.

The interaction between target type and congruency was significant, *F*(2, 233.22) = 147.68, *p* < .0001, η2p = .559. Contrast analyses, showed differences between faces and arrows in congruent, *t*(24.0) = 7.77, *p* < .0001, *d* = 1.438, and incongruent conditions, *t*(24.0) = 4.22, *p* = .0010, *d* = .781. Similarly, significant differences were found between eyes and arrows in congruent, *t*(19.2) = 9.00, *p* < .0001, *d* = 1.464, and incongruent trials, *t*(19.2) = 5.38, *p* < .0001, *d* = .875. Both social targets led to slower RTs in both congruency conditions compared with arrows (Table 3). In contrast, no significant differences were observed between faces and eyes either in congruent or in incongruent trials.

**TABLE 3:** Estimated marginal means of reaction times by target type and congruency.

Target type	Congruent		Incongruent	
	EMM‐RT	95% CIs	EMM‐RT	95% CIs
Face	624 (13.0)	[597, 651]	595 (13.0)	[569, 622]
Eyes	627 (11.5)	[603, 650]	604 (11.5)	[580, 628]
Arrows	493 (10.9)	[470, 515]	524 (10.9)	[502, 546]

*Note*: The column EMM‐RT shows the estimated marginal means of RT in milliseconds, with the standard error in parentheses. The 95% confidence interval for the EMM‐RT are provided in brackets.

**FIGURE 1:**
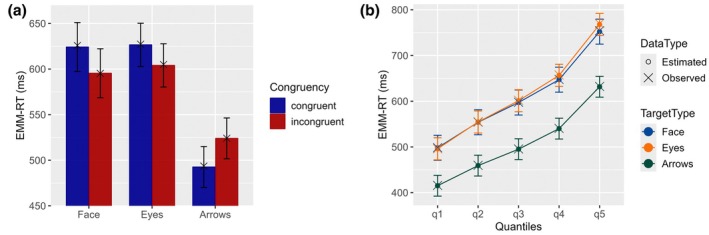
Interactions in the RT model. (a) Bar plot of congruency by target type; (b) CDF plot of estimated marginal means of reaction time (EMM‐RT) by quantile at .1, .3, .5, .7, .9 probabilities. The bars represent the 95% confidence interval for the EMM‐RT. Observed values are denoted by ‘X' and estimated values by ‘O'.

Within the same interaction, contrast analyses between congruency levels by target type showed the reversion for both, face targets, *t*(190) = 9.26, *p* < .0001, *d* = .314, and eyes targets, *t*(227) = 8.05, *p* < .0001, *d* = .246, where incongruent trials were faster than congruent trials (Figure 1a). In contrast, arrows exhibited the SCE, *t*(192) = −12.16, *p* < .0001, *d* = −.343, with faster responses in congruent compared with incongruent trials.

The interaction between target type and quantile (Figure 1b) was also significant, *F*(8, 233.22) = 12.86, *p* < .0001, η2p = .306. Contrast analyses by quantile revealed no differences between face and eyes targets across the distribution. Moreover, both face and eye stimuli were significantly slower than arrows at all five quantile points, although the difference increased across quantiles.

The RT model replicates the SCE with arrows and the RCE with face and eye targets observed in previous studies (Chacón‐Candia et al., 2024; Ishikawa et al., 2021; Marotta et al., 2018; Tanaka et al., 2023). Additionally, non‐social targets elicited faster RTs than social targets, consistent with prior findings (Bonventre & Marotta, 2023; Dalmaso et al., 2023; Ishikawa et al., 2021; Narganes‐Pineda et al., 2022). This difference between social and non‐social targets also grew larger with increasing quantiles (Figure 1b).

However, no significant differences were found between face and eye targets, despite the expectation that faces would consistently yield slower RTs due to their more complex perceptual configuration. This pattern across quantiles suggests that faces do not necessarily require additional processing time. Moreover, these equivalent response times between faces and isolated eyes appear to be unaffected by congruency, indicating that congruent (incongruent) faces elicit responses at equivalent times that congruent (incongruent) eyes. If background complexity influenced RTs, faces would consistently show slower responses across studies, either in terms of congruency effects or overall RTs. Alternatively, if both social targets share similar components – whether in terms of social significance or perceptual processing mechanisms related to extracting relevant information (see General Discussion) – conflict inhibition processes may integrate these components with spatial information codes, leading to similar attentional orienting patterns, in a way that results in equivalent RTs.

### Delta model and trend analyses

The ANOVA on the delta model revealed a main effect of target type, *F*(2, 24.72) = 97.43, *p* < .0001, η2p = .887. Both social targets differed significantly from arrows, which exhibited a positive congruency effect (*∆*_arrows_ = 30.8, SE = 3.39). Additionally, no significant difference was found between face and eyes (*p* = .3519), with both showing negative delta values (∆_face_ = −29.7, SE = 4.08 & *∆*_eyes_ = −22.0, SE = 3.62). There was also a main effect of quantile, *F*(4, 92.34) = 23.08, *p* < .0001, η2p = .500.

**FIGURE 2:**
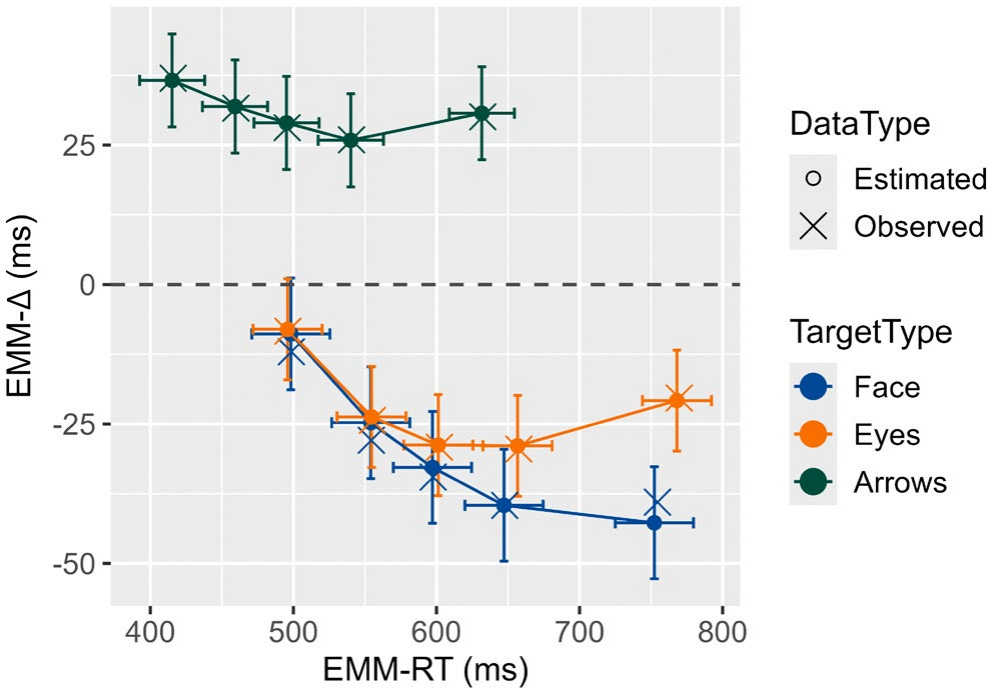
Delta plots derived from the delta model. The *y*‐axis displays the estimated marginal means of the delta function (EMM‐∆), with bars representing the 95% confidence intervals. The *x*‐axis shows the estimated marginal means of reaction time (EMM‐RT) at .1, .3, .5, .7, .9 probabilities, with the bars representing the 95% confidence interval for the EMM‐RT. Observed values are represented by ‘X’ and estimated values by ‘O’.

The model revealed a significant two‐way interaction between target type and quantile, *F*(8, 92.01) = 3.99, *p* = .0004, η2p = .258. Importantly, this interaction was also found in the quartile and decile models and when employing the non‐trimmed dataset, with differences between social and non‐social targets remaining consistent across the distribution. Notably, no differences were observed in the fastest responses between faces and eyes; the only significant difference emerged at the fifth quantile, *t*(50.7) = −3.26, *p* = .0060, *d* = −.400, with faces showing a more negative congruency effect (Figure 2). Similar patterns were observed in the quartile and decile models, with marginal differences between social targets at the fourth quartile and significant effects at the ninth and tenth deciles, respectively (see Appendix S1).

Contrast analyses against zero by quantile showed significant SCEs for arrows across all quantiles (*t* = 6.23 to 8.82, *p* < .0001, *d* = .473 to .669), a finding that held in both quartile and decile models – indicating a SCE from the fastest to the slowest responses. In contrast, the RCE for social targets emerged from the second quantile onward, with faces yielding *t*‐values from −8.57 to −4.97, *p* < .0001, *d* = −.781 to −.452, and eyes from −6.40 to −4.60, *p* < .0001, *d* = −.528 to −.380. In the quartile model, the RCE was evident from the first quartile for both eyes and faces, although this effect was partly dependent on the probability chosen to construct the dataset (see quartile‐B model in Appendix S1). The decile model, however, showed significant reversion from decile 2 to 10, with no conflict effect observed at decile one. Overall, yielding similar and equivalent conflict effect sizes for both social targets within the fastest and intermediate response latencies.

Polynomial trend analysis revealed a significant negative linear coefficient for faces (coeff. = −.200 [−.23, −.17], *p* < .0001), and for eyes (coeff. = − .092 [−.13, −.06], *p* < .0001), along with a significant positive quadratic term for all target types: faces (coeff. = − .0003 [.0002, .0005], *p* < .0001), eyes (coeff. = − .0003 [.0002, .0004], *p* < .0001), and arrows (coeff. = − .0003 [.0002, .0004], *p* < .0001). This pattern remained consistent in the non‐trimmed dataset for social targets, whereas both coefficients were non‐significant for arrows (see Table S3 in the Supplemental Material). The quartile model consistently showed negative linear and positive quadratic terms for all targets, and the decile model confirmed negative linear terms for faces and eyes along with significant quadratic terms across all three targets. These findings suggest that trend analysis for arrows is sensitive to the trimming procedure and quantile selection, while faces and eyes maintain a stable trend.

Additionally, faces displayed a significantly more negative linear coefficient than eyes, diff. = −0.108, SE = 0.0197, *t*(5714) = −5.48, *p* < .0001, and arrows, diff. = −0.192, SE = 0.0159, *t*(5755) = −12.11, *p* < .0001. Eyes also showed a more negative slope than arrows, diff. = −0.085, SE = 0.0166, *t*(5552) = −5.09, *p* < .0001. These differences were stable across the different models (see Appendix S1). These results indicate that, as response latencies increase, the negative trend becomes more pronounced for face targets.

The delta model showed a stable SCE for arrows across the distribution, regardless of the number of quantiles used. However, as indicated by the trend analysis, the reduction in conflict effects was not uniform across quantiles, differing from the typical patterns observed in other location‐based conflict tasks, such as the Simon or Simon‐like tasks (De Jong et al., 1994; Luo & Proctor, 2017, 2019). Similarly, Tanaka et al. (2025) found that the SCE with arrows persisted throughout the distribution in experiment 1, but in the combined and supplemental analyses, no significant spatial Stroop effect appeared in the last data point. However, comparisons between points 3 and 4 (called bins by the author) did not reach significance, suggesting stabilization in the reduction of the conflict effect.

In spatial Stroop tasks, the reduction in the congruency effects is often less pronounced and seems to affect the fastest and middle response latencies (Castel et al., 2007, Figure 2, experiment 1A; Juncos‐Rabadán et al., 2008, see *t*‐tests performed with the youngest group).^2^ This contrasts with the visual Simon effect, where conflict reduction can extend to the slowest responses (Luo & Proctor, 2019, experiment 1A) and sometimes even eliminates the conflict effect at slower RTs (Mittelstädt & Miller, 2020, experiment 1; Zhong et al., 2020). The similarities in faster responses may stem from both tasks eliciting conflict related to the irrelevant spatial location of the stimuli. Conversely, the differences may reflect distinct underlying mechanisms – possibly due to additional dimensional overlaps between task‐relevant and ‐irrelevant stimulus features, as well as between task‐relevant information and response in the spatial Stroop, which are absent in the Simon task (Kornblum, 1992; Viviani, Visalli, Montefinese, et al., 2024). Previous research has shown that these task‐related differences yield distinct conflict patterns likely due to variations in their underlying temporal dynamic, ultimately modulating delta plot shapes (Egner, 2008; Funes et al., 2010; Liu et al., 2004; Luo & Proctor, 2017, 2019; Pratte et al., 2010; Scerrati et al., 2017; Smith & Ulrich, 2025; Torres‐Quesada et al., 2014, 2022; Ulrich et al., 2015).

For social targets, the RCE was observed from the second quantile onward (or from the second decile in the decile model) and from the first quartile in the quartile model – consistent with Tanaka et al.'s (2025) findings on gaze. The different patterns observed in the fastest responses between the quartile model and other models seem to depend on the chosen quantile probabilities (see Appendix S1). Nonetheless, consistent findings between models indicate that within the fastest responses, the conflict effect is minimal and does not reach significance. This supports the idea that spatial interference does diminish before the RCE can emerge (Román‐Caballero et al., 2021a, 2021b; Tanaka et al., 2024, 2025).

Moreover, differences between faces and eyes were not evident until the slowest responses, which have implications for background segregation. If extracting relevant information from the background is necessary for the RCE, it should influence the fastest or middle response latencies – particularly for faces, which have greater perceptual complexity. Alternatively, background segregation may be executed similarly for both social stimuli, potentially reflecting processes linked to gaze perception rather than strict segregation mechanisms (Kobayashi & Kohshima, 1997; Stephenson et al., 2021). Some evidence highlights the crucial role of the eyes in early face processing (Itier & Preston, 2018; Nemrodov et al., 2014; Nemrodov & Itier, 2011), which could facilitate the extraction of task‐relevant information at similar times for faces and isolated eyes before attentional orienting is triggered.

Following the initial reduction of the conflict effect, the reversion emerges and then amplifies until reaching a peak, as indicated by the trend analysis (i.e., quadratic trend). This increase in the reversion effect is reflected by a negative‐going delta plot (Figure 2). A similar pattern was reported by Tanaka et al. (2025, combined analysis), who found an initial negative‐going delta plot followed by a reduction in the RCE when examining eye‐gaze stimuli (although see results of the experiment 1).

Negative‐going delta plots commonly appear in visual Simon tasks (Ambrosi et al., 2019; De Jong et al., 1994; Miles & Proctor, 2012; Ridderinkhof, 2002a; van Campen et al., 2014) and can even reveal reversed conflict effects during slower responses – particularly under manipulations of motor response compatibility (De Jong et al., 1994) or proportion compatibility (Ridderinkhof, 2002a, 2002b; with Flanker task see Hübner & Töbel, 2019).

When irrelevant information is spatial (i.e., stimuli are presented on the left or right), these patterns are typically explained by active control models, which propose that the irrelevant dimension is actively suppressed by a flexible control mechanism whose onset and strength may vary based on task characteristics and individual differences (Hübner & Töbel, 2019; Ridderinkhof, 2002a, 2002b; Ulrich et al., 2015; van Campen et al., 2014). Alternatively, passive‐decay models assume that irrelevant information simply fades over time (Hommel, 1993; Mittelstädt & Miller, 2020; Pratte et al., 2010; van den Wildenberg et al., 2010). However, under both accounts, conflict is typically stronger in faster responses and then diminishes over time, resulting in negative‐going delta plots (or inverted U‐shaped delta plots, for example, Davranche & McMorris, 2009; Ulrich et al., 2015). Hence, in addition to reflecting the strength and onset of control mechanisms, the shape of the delta plot may also indicate the relative speed at which task‐irrelevant versus task‐relevant information is processed (Mackenzie et al., 2022; Miller & Schwarz, 2021; Mittelstädt et al., 2023).

Overall, this interplay among control mechanisms, processing speed, and the dimensional overlap characteristic of the spatial Stroop likely modulates both the observed reversion and the delta plot trend. As mentioned previously, these task‐related factors might produce outcomes that resemble the Simon effect but also introduce unique patterns. For instance, reversed conflict effects with non‐social targets have been observed (Chen et al., 2022, experiment 2; Román‐Caballero et al., 2021a, experiment 1), along with negative‐going delta plots in which the reversion emerged during slower responses (Tanaka et al., 2025) – a phenomenon linked to increased difficulty in extracting relevant information (see the dual‐stage hypothesis; Tanaka et al., 2024, 2025). In contrast, when gaze stimuli are used, the reversion appears as early as the fastest responses (i.e., quantile two) and remains larger at mid‐range response times, suggesting that the RCE develops earlier for social stimuli, potentially due to their social relevance or distinctive perceptual features.

## GENERAL DISCUSSION

This study investigated attentional orienting differences between social and non‐social stimuli, focusing on faces and eyes as social targets. A distributional approach was used, employing CDF analyses and distributional and trend analyses of delta functions. Data from 11 studies (Table 1) were combined, yielding a sample of 705 participants. Three models were applied: the RT model (from the CDF dataset) to compare RT distributions by target type and congruency; the delta model to examine conflict effects across quantiles; and the Trend model to identify specific trends in delta functions using orthogonal polynomial contrast analyses.

In summary, non‐social stimuli consistently produce SCE, with quicker responses in congruent trials across all quantiles. In contrast, social stimuli such as faces and eyes displayed an RCE, where faster responses are observed in incongruent trials starting from the second quantile onward, following a quadratic trend. This early dissociation suggests a more complex processing dynamic for social stimuli, possibly due to their inherent complexity and the social cognitive mechanisms they engage.

On the other hand, the absence of differences between faces and eyes in the CDF results and in the fastest and middle‐range responses of the delta function suggests that both social targets undergo similar early processing in terms of overall RTs and conflict inhibition patterns. This early phase of processing is likely influenced by the eye region, which plays a crucial role in how faces are processed (Bentin et al., 1996; Itier et al., 2007; Itier & Preston, 2018; Nemrodov & Itier, 2011; Rousselet et al., 2014). The distinct white sclera of human eyes enhances the detection of small gaze shifts (Calder et al., 2008; Kobayashi & Kohshima, 1997, 2001), allowing for rapid attentional capture by both eyes and full faces, making their processing at this stage equivalent. Prior research suggests that attention is automatically drawn to biologically and socially significant stimuli, such as faces (Eastwood et al., 2001; Ro et al., 2007), enhancing attentional capture (Langton et al., 2008; Morrisey et al., 2019; Theeuwes & Van der Stigchel, 2006).

Within the dual‐route framework, this process aligns with theories like the activation–suppression race model (Miller & Schwarz, 2021), which suggests a competition between the suppression of irrelevant activation and the identification of relevant information prior the selection and motor execution of a response. In a scenario where eye‐gaze and face stimuli are processed, this means that if relevant information is processed faster and/or if the influence of irrelevant spatial information decays or is suppressed by the time directional cues trigger a response, its impact is diminished. This could explain the absence of significant differences in early responses between eye and face stimuli.

However, these early perceptual and attentional mechanisms do not fully account for the subsequent increase in the RCE or for the more pronounced effect observed for faces compared with eyes in slower responses.

From a social processing perspective, intermediate and slower responses might allow higher order social processes – such as eye contact (Cañadas & Lupiáñez, 2012; Marotta et al., 2018; Ponce et al., 2024), joint attention (Edwards et al., 2020), or joint distraction episodes (Hemmerich et al., 2022). Full faces which inherently contain more complex and nuanced information, like emotional expressions, intentions, gender, or dominance (Bernstein et al., 2007; Haxby et al., 2000; Leopold & Rhodes, 2010), may therefore facilitate a more prolonged RCE. If facial stimuli engage attention for longer periods than simpler cues like isolated eyes (Langton et al., 2008; Theeuwes & Van der Stigchel, 2006), the temporal window for the emergence of a ‘looking vector’ – a socially meaningful attentional signal that operates beyond basic directional cueing (Hemmerich et al., 2022) – increases. This extended engagement may amplify the peak of the reversion, as reflected in the delta plot, when compared with the effects observed with cropped‐eyes. Indeed, prior studies using full‐face cues with additional social features – such as emotional expression (Marotta et al., 2022; Tanaka et al., 2023; Torres‐Marín et al., 2017) or social familiarity (Ishikawa et al., 2024) – have shown notable modulations of spatial conflict effects.

These findings suggest that the ‘looking vector’ may support privileged access to socially meaningful spatial information, facilitating enhanced attentional selection and faster responses towards the spatial location that the stimulus appears to be ‘looking at’ (Hemmerich et al., 2022). Originally introduced within the framework of joint distraction, the looking vector may also reflect joint attention episodes or implicit eye contact, depending on task context and stimulus configuration. Instead of treating these theoretical accounts as mutually exclusive, we interpret the looking vector as a flexible mechanism supporting several higher order social processes. This perspective aligns with models of gaze perception and social cognition that emphasize dynamic interactions between bottom‐up and top‐down systems (Hadders‐Algra, 2022; Yang et al., 2015), which ultimately shape attentional dynamics over time (Stephenson et al., 2021).

Alternatively, social and task‐related accounts may not represent competing explanations. Instead, the mechanisms proposed by both perspectives could operate together, dynamically contributing to the emergence and modulation of the RCE (Ponce et al., 2024; Román‐Caballero et al., 2021a, 2021b; Tanaka et al., 2024, 2025). Social features may enhance attentional engagement and the salience of directional cues (Román‐Caballero et al., 2021a, 2021b; Tanaka et al., 2024), while the structural characteristics of the spatial Stroop task determine when and how interference arises and is resolved (Ponce et al., 2024; Tanaka et al., 2025).

Building on this interplay, we suggest that social processes might be modulated by the inherent structure of the spatial Stroop task at both stimulus and response levels (Kornblum, 1992, 1994; Viviani, Visalli, Finos, et al., 2024; Viviani, Visalli, Montefinese, et al., 2024). At the stimulus level, stimulus–stimulus (S–S) overlap arises when both relevant and irrelevant dimensions contain spatial information (Luo & Proctor, 2017, 2019; Torres‐Quesada et al., 2022). For instance, eyes on the left side of the screen looking right produce a congruency effect tied to processes at the stimulus identification stage (Kornblum, 1992, 1994), reflecting a different time window for conflict processing compared with response level conflict (Frühholz et al., 2011), and suggesting a perceptual basis (Kornblum et al., 1999; Scerrati et al., 2017). As discussed in previous paragraphs, early extraction of gaze information during stimulus identification stage may account for the delta plot pattern observed in the fastest responses.

In addition, the spatial Stroop involves stimulus–response (S–R) overlap, where both relevant and irrelevant stimulus dimensions influence the response. Research comparing S–S and S–R overlap points to distinct and independent mechanisms for handling conflicting information and inhibiting responses, potentially affecting different segments of the response latency distribution (Correa et al., 2010; Egner, 2008; Frühholz et al., 2011; Li et al., 2014; Luo & Proctor, 2017, 2019; Scerrati et al., 2017; Torres‐Quesada et al., 2014, 2022). In particular, when task‐irrelevant information overlaps with the response dimension, S–R conflict is thought to emerge during the response selection stage through the superimposition of relevant and irrelevant information processing and a response capture mechanism favouring the irrelevant information (De Jong et al., 1994; Lu & Proctor, 1995; Scerrati et al., 2017; Torres‐Quesada et al., 2022; van Campen et al., 2014; van Campen et al., 2018). Interestingly, it has been observed that conflict processing and inhibition can persist into response initiation and motor execution (Miller & Roüast, 2016; Scorolli et al., 2015; Servant et al., 2016). This is particularly relevant given that the spatial Stroop also involves overlapping between task‐relevant information and the response dimension, which might contribute further to the resultant conflict effect (Zhang & Kornblum, 1998), by modulating response capture.

Within this S–R framework, dual‐route models propose that an automatic route processes stimulus onset independently of the S–R mapping instructions, with response capture reflecting the susceptibility of selecting a response based on irrelevant spatial location (Cohen et al., 1990; De Jong et al., 1994; Hommel, 1993; Hübner et al., 2010; Miller & Schwarz, 2021; Mittelstädt et al., 2023; Ridderinkhof, 2002a, 2002b; Ulrich et al., 2015; van Campen et al., 2014). For example, when a stimulus appears on the right side, it automatically triggers a rightward response and right‐hand motor preparation (Torres‐Quesada et al., 2022; van Campen et al., 2014; van Campen et al., 2018), even if the correct response is to the left. At the response selection stage, this automatic activation is held in check by the controlled route (Ridderinkhof, 2002a, 2002b; Ulrich et al., 2015; van Campen et al., 2014). When relevant and irrelevant information conflict (i.e., incongruent trials), additional control processes are engaged to resolve the discrepancy, leading to slower RTs and increased error rates.

The overall impact of information carried via the automatic route (i.e., irrelevant spatial location) depends on both the onset timing and suppression strength of task‐irrelevant activation (Ridderinkhof, 2002a, 2002b; Ulrich et al., 2015), as well as any enhancement of task‐relevant processing (Mittelstädt et al., 2023; Mittelstädt & Miller, 2020; Servant et al., 2016). These suppression mechanisms are essential for counteracting response capture (Ridderinkhof, 2002a, 2002b; van Campen et al., 2014; van Campen et al., 2018), which – as noted – is triggered by the irrelevant spatial location. Importantly, this interaction between automatic and controlled processes can be further modulated by specific stimulus features (Ellinghaus et al., 2024; Mittelstädt & Miller, 2020), leading to different delta function patterns.

In this regard, social targets may further modulate the balance between automatic activation and control mechanisms. Prior work suggests that social stimuli may recruit additional resources – either due to their social salience (Hemmerich et al., 2022; Marotta et al., 2018, 2019; Román‐Caballero et al., 2021a, 2021b) or their perceptual complexity, which may hinder to some extent gaze direction extraction (Tanaka et al., 2024, 2025). These factors may ultimately influence the strength of response capture and/or the demands on control mechanisms. For instance, conditional accuracy function (CAF) analyses (Ponce et al., 2024) showed that social targets elicit distinctive response capture mechanisms. Specifically, these targets not only produce a response capture towards the irrelevant information – reflected by lower accuracy in the first bin compared with later bins in incongruent trials (for a similar interpretation of response capture with CAF, see van Campen et al., 2014) – but also reverse the pattern typically observed with non‐social targets (Torres‐Quesada et al., 2022; van Campen et al., 2014; van Campen et al., 2018) with even lower accuracy for congruent trials in the initial bins. Notably, this CAF reversion was more apparent for faces than isolated eyes, and the effect was amplified when faces with emotional expressions were included (see model MB and MC in Ponce et al., 2024).

This suggests that relevant information from social targets may also be processed via the automatic route to some extent and/or that control mechanisms are engaged more rapidly, either by inhibiting irrelevant information earlier (Ponce et al., 2024; Tanaka et al., 2025), or by enhancing the processing of relevant information.

If social targets require stronger inhibition for successful response selection (Tanaka et al., 2024, 2025), and if such inhibition takes time to develop (Ridderinkhof, 2002a, 2002b), this would primarily impact middle‐range and slower responses, where the peak of the RCE was observed. This would model the dynamic of the RCE, for instance, following a similar description of Tanaka's second stage (Tanaka et al., 2024, 2025), in incongruent trials, the incorrect response (and its motor preparation) may already be inhibited by the time the relevant information is processed, allowing the correct response to proceed faster (and accurately). Conversely, in congruent trials the automatic response capture remains inhibited until the controlled mechanism selects the correct response; once selected, any remaining inhibition must dissipate before motor execution, leading to slower overall RTs (while the inhibition is not decreased the error rates increase).

Moreover, face stimuli, with their greater perceptual complexity compared with isolated eyes, may further intensify this mechanism – as supported by the CAF results in Ponce et al. (2024) – resulting in a more pronounced RCE, particularly evident in the slower responses of the delta function. The stronger automatic activation elicited by full‐face stimuli would demand not only earlier onset but also greater strength of inhibition. If this inhibition persists even after the correct response is selected, it could lead to longer delays before execution in congruent trials, thereby amplifying the RCE, while still maintaining a similar overall delta shape compared with eyes (as indicated by the trend analysis). These dynamics align with variability observed in location‐based conflict tasks (Luo & Proctor, 2019; Mittelstädt et al., 2023; Ridderinkhof, 2002a, 2002b) and in diffusion model parameters (Luo & Proctor, 2020; Ulrich et al., 2015), particularly with respect to the amplitude and onset timing of the automatic mechanism.

For consideration, this interpretation of the delta function patterns builds on previous findings showing that both active control mechanisms and processing speed (De Jong et al., 1994; Mackenzie et al., 2022; Mittelstädt et al., 2023; Mittelstädt & Miller, 2020; Ridderinkhof, 2002a, 2002b), along with dimensional overlap (Luo & Proctor, 2017, 2019; Scerrati et al., 2017; Torres‐Quesada et al., 2014, 2022), contribute to shaping delta plot dynamics. This view is consistent with the idea that the Stroop effect involves multiple loci and stages of processing (Augustinova et al., 2018; De Houwer, 2003; Parris et al., 2022; Viviani, Visalli, Finos, et al., 2024; Viviani, Visalli, Montefinese, et al., 2024; Zhang & Kornblum, 1998).

Nonetheless, it is important to acknowledge that the current methods do not permit a direct mapping of delta patterns onto more specific discrete cognitive stages. While delta functions provide informative insights into temporal dynamics – and have been widely used to support dual‐route models in conflict tasks (Ambrosi et al., 2019; Balota & Yap, 2011; Hübner & Töbel, 2019; Miller & Schwarz, 2021; Mittelstädt & Miller, 2020; Smith & Ulrich, 2025) – their interpretative resolution remains limited. For more detailed hypothesis testing, we recommend complementing this approach with parameter estimation methods (Rouder & Speckman, 2004), such as computational modelling using the diffusion model for conflict tasks (DMC; Ulrich et al., 2015).

Additionally, we develop this proposal under the assumption that these patterns reflect a mechanism specific to gaze processing within the spatial Stroop task (Ponce et al., 2024; Tanaka et al., 2025). Nevertheless, Tanaka et al. (2024) observed a reversion with tongues embedded in faces by the second data point in a four‐point delta function (Tanaka et al., 2024, Supplemental Material), although no conflict effect was elicited in the first point of the distribution – unlike the pattern seen with gaze (first experiment and combined analysis). Similarly, our results revealed the RCE emerging as early as the second quantile in a five‐point delta function, suggesting that gaze might trigger reversion sooner, following a different initial trajectory. However, because tongues were also embedded in faces, they likely conveyed face‐related information at early stages, eliciting similar patterns. The mouth region plays a key role in face processing (Itier & Preston, 2018; Luo et al., 2013), and the tongue can cue attention similarly to gaze (Downing et al., 2004, experiment 1) or express emotion (Rozin et al., 1994), potentially creating comparable patterns.

On the other hand, the RCE has also been observed with non‐social targets (Chen et al., 2022, experiment 2; Román‐Caballero et al., 2021a, experiment 1; Tanaka et al., 2025), indicating that it is not exclusive to gaze mechanisms. Further exploration of subtle differences in temporal conflict elicitation and inhibition may reveal unique dynamics attributable to gaze versus other types of stimuli. Such investigations could help distinguish gaze‐driven patterns from those shaped by general task dynamics or perceptual factors.

## CONCLUSION

This study reveals that social and non‐social stimuli elicit distinct temporal dynamics in spatial conflict resolution. While arrows produced a SCE across the distribution, gaze stimuli elicited a RCE from the early quantiles of the distribution onward – highlighting their unique influence on attentional control. Among social stimuli, although faces are perceptually more complex than isolated eyes, both produced comparable RTs and similar delta function patterns in early responses – suggesting that gaze direction is extracted at similar stages, likely due to shared attention to the eye region. However, delta plots diverged in slower responses in which faces produced a larger RCE than eyes, indicating that either their social nature or added perceptual complexity amplifies response‐related control mechanisms over time. These findings highlight how social stimuli modulate conflict resolution not only through initial gaze extraction but also via late‐stage processes, offering new insight into how gaze cues differ from arrows in shaping spatial attention under conflict.

## AUTHOR CONTRIBUTIONS

**Renato Ponce:** Conceptualization; data curation; formal analysis; methodology; software; visualization; writing – original draft; writing – review and editing; investigation. **Juan Lupiáñez:** Conceptualization; data curation; funding acquisition; methodology; project administration; supervision; validation; writing – review and editing; resources; investigation. **Carlos González‐García:** Data curation; methodology; validation. **Maria Casagrande:** Funding acquisition; supervision. **Andrea Marotta:** Conceptualization; data curation; funding acquisition; methodology; project administration; supervision; validation; writing – review and editing; resources; investigation.

## CONFLICT OF INTEREST STATEMENT

None of the authors have any financial interest or conflict of interest.

## Supporting information


Appendix S1.

## Data Availability

The data, the Appendix S1, and the script of the analysis that support the findings of this study are openly available in OSF at https://osf.io/twbs3/.

## Footnotes

^1^
The term ‘background complexity’ is used here as defined in Román‐Caballero et al. (2021a, 2021b) and Tanaka et al. (2024, 2025). In this context, a complex perceptual background refers to either coloured geometric rectangular patterns or face stimuli. In both cases, the authors demonstrated that arrows and eyes embedded within such complex backgrounds—geometric or facial—modulate the resulting conflict effect.

^2^
In Castel et al. (2007) and Juncos‐Rabadán et al. (2008), tasks involving a Type‐8 ensemble are described in terms of a Simon effect. For consistency, however, we refer to them here as spatial Stroop tasks, as they employ lateralized arrows with motor responses—thus creating overlap among relevant direction and irrelevant physical position, as well as among direction and motor response, and location and motor response (Kornblum, 1992; Viviani, Visalli, Montefinese, et al., 2024). These tasks match those used in the current analyses.
