# Supplementary material for: Investigating the gaze‐driven reversed congruency effect in the spatial Stroop task: A distributional approach
Source: Br J Psychol. 2025 Jun 20;116(4):1107–27. doi: 10.1111/bjop.70004 (PMC12514322; doi:10.1111/bjop.70004)
Supplement: Supplementary file 1 — Appendix S1. [file BJOP-116-1107-s001.docx]

Supplemental Material

*Table of contents*

[*Trimming procedure* 1](#_Toc193275509)

[*Grouping structure & stepwise analysis results* 2](#_Toc193275510)

[*Non-trimmed, quartile and decile models* 4](#_Toc193275511)

[*Trend analyses* 5](#_Toc193275512)

# *Trimming procedure*

For the trimming procedure, we adopted the cutoff thresholds used in most original studies, discarding reaction times below 200ms and above 1300ms (Table 1). This approach was consistently applied across all studies, including Dalmaso et al. (2023), despite their original use of a 3 standard deviation (SD) trimming method. The analyses included only correct responses.

| **Table 1.** Error rates, remaining trials and trials removed by study | | | |
| --- | --- | --- | --- |
| **Study** | **Remaining trials (%)** | **Incorrect trials (%)** | **Trials trimmed (%)** |
| Torres-Marín et al. (2017), experiments 1 / 2 | 94.56% / 95.05% | 5.44% / 4.95% | .01% (*<200*), .75% (*>1300*) /  .00% (*<200*), .92% (*>1300*) |
| Marotta et al. (2018) | 96.85% | 3.15% | .01% (*<200*), .25% (*>1300*) |
| Marotta & Lupiáñez (2018) | 97.97% | 2.03% | .00% (*<200*), .38% (*>1300*) |
| Marotta et al. (2019) | 96.79% | 3.21% | .00% (*<200*), .38% (*>1300*) |
| Hemmerich et al. (2022), experiments 1 / 2 | 97.77% / 95.55% | 2.23% / 4.45% | .00% (*<200*), .64% (*>1300*) /  .00% (*<200*), .52% (*>1300*) |
| Narganes-Pineda et al. (2022), experiment 1 | 97.34% | 2.66% | .00% (*<200*), .52% (*>1300*) |
| Marotta et al. (2022) | 94.63% | 5.37% | .01% (*<200),* .82% (*>1300*) |
| Bonventre & Marotta (2023), experiments 1 / 2 | 97.75% / 96.92% | 2.25% / 3.08% | .00% (*<200*), .37% (*>1300*) /  .00% (*<200*), .55% (*>1300*) |
| Ishikawa et al. (2024), experiments 1 / 3 | 96.01% / 95.86% | 3.99% / 4.14% | .12% (*<200*), .65% (*>1300*) /  .30% (*<200*), .27% (*>1300*) |
| Tanaka et al. (2023), experiments 1A / 1B | 94.87% / 95.8% | 5.13% / 4.20% | .04% (*<200*), .59% (*>1300*) /  .00% (*<200*), .09% (*>1300*) |
| Dalmaso et al. (2023) | 97.31% | 2.69% | .00% (*<200*), .07% (*>1300*) |
| ***Note.*** For studies with multiple experiments, the reported values are separated by a slash. The column ‘Remaining trials’ shows the percentage of correct responses left after applying the trimming procedure and removing incorrect trials. The reported incorrect trials were calculated post-trimming. The column ‘Trials trimmed’ indicates the percentage of correct trials that were faster than 200ms (<200) or slower than 1300ms (>1300). | | | |

Additionally, the same analyses were conducted after excluding trials without a response (i.e., employing the non-trimmed dataset). Both the RT and Delta models remained consistent with the overall analysis. In contrast, the Trend model analyses showed consistent patterns for face and eye targets, but significant changes were observed with arrow targets (see Trend analysis section), which are mentioned in the manuscript. The non-trimmed datasets and script are available on the OSF webpage (see Data Availability Statement).

# *Grouping structure & stepwise analysis results*

The best-fitting models were selected using a stepwise approach with the ‘step’ function from the ‘lmerTest’ R package (Kuznetsova et al., 2017) on the RT and Delta models. The RT model initially included a three-way interaction between target type, congruency and quantile (QQ), while the Delta model included a two-way interaction between target type and quantile (QQ). Random effects reflected the grouping structure, following Ponce et al. (2024), using crossed effects syntax for implicit nesting (e.g., Schielzeth & Nakagawa, 2013), while also considering strategies to improve model fit (Barr, 2013; Meteyard & Davies, 2020). Participants were nested within studies (1|Studies:id), with target type treated as study-specific (*1|Study:TargetType*). The unique distributional structure of each study was also accounted for: the RT model included congruency and quantile factors (*1|Study:TargetType:Congruency:QQ*), and the Delta model included quantile factor (*1|Study:TargetType:QQ*). This approach allowed for more flexible modeling, capturing both individual and target-type variations within studies, as well as distributional differences within studies.

The stepwise process used backward elimination, sequentially removing effects, starting with random effects and then fixed effects, while adhering to the principle of marginality (McCullagh & Nelder, 1989). P-values for fixed effects are derived from an F-test using Satterthwaite’s approximation, while random effects p-values were calculated using likelihood ratio tests. In the RT model, the three-way interaction was not significant, leading to a simplified model focusing on two-way interactions. The Delta model showed a significant interaction between target type and quantile. In both models, all random grouping structures yielded significant results.

| **Table 2.** Stepwise analyses results | | | |
| --- | --- | --- | --- |
| **RT model** | | | |
| **Random parameters** | **logLik** | **LRT** | **p** |
| (1:Study:id) | -71 665 | 9 825.8 | < .0001 |
| (1\|Study:TargetType) | -66 862 | 219.3 | < .0001 |
| (1\|Study:TargetType:Congruency:QQ) | -66 842 | 180.5 | < .0001 |
| **Fixed parametersR** | **Sum Sq** | **F** | **p** |
| TargetType * Congruency * QQ | 13 477 | .63 | .7526 |
| TargetType * Congruency | 790 414 | 147.68 | < .0001 |
| TargetType * QQ | 275 224 | 12.86 | < .0001 |
| Congruency * QQ | 45 469 | 4.25 | .0025 |
| **Delta model** | | | |
| **Random parameters** | **logLik** | **LRT** | **p** |
| (1:Study:id) | -32 988 | 855.92 | < .0001 |
| (1\|Study:TargetType) | -32 579 | 36.85 | < .0001 |
| (1\|Study:TargetType:QQ) | -32 565 | 10.28 | .0013 |
| **Fixed parameters** | **Sum Sq** | **F** | **p** |
| TargetType * Congruency * QQ | 68 580 | 3.99 | .0004 |
| ***Note.*** the logLik column represents the likelihood function, the LRT provides the likelihood ratio test estimates, and the Sum Sq shows the sum of squares. | | | |

# *Non-trimmed, quartile and decile models*

The delta model fitted with the non-trimmed dataset, as well as the quartile and decile models, followed the same protocols used in the main delta model reported in the manuscript. In all cases, the two-way interaction between target type and quantile was significant, indicating consistent overall patterns.

The quartile model used four data points at probabilities *.2, .4, .6*, and *.8* (e.g., Castel et al., 2007), employing equally space probabilities (e.g., Tanaka et al., 2025). Additionally, a modified quartile model (quartile-B) was tested at probabilities *.1, .3, .7* and *.9* to emphasize the tails of the distribution—similar to the approach used in the main delta model (i.e., five quantiles).

| **A)**  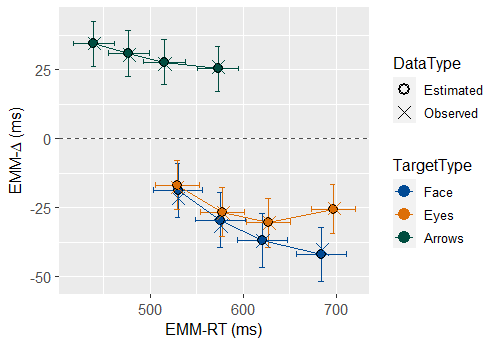 | **B)**  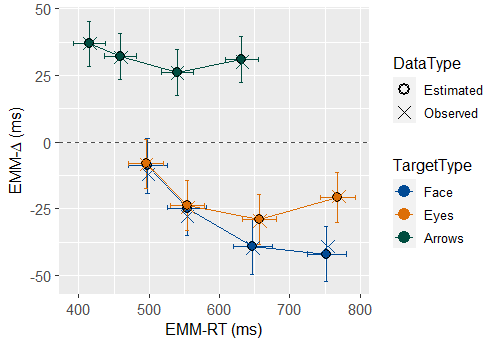 |
| --- | --- |
| **Figure 1.** Delta plots of quartile model is shown in A) and its modified version, quartile-B, is shown in B). The y-axis displays the estimated marginal means of the delta function (EMM-∆), with bars representing the 95% confidence intervals. The x-axis shows the estimated marginal means of reaction time (EMM-RT), with the bars representing the 95% confidence interval for the EMM-RT. Observed values are represented by ‘X’ and estimated values by ‘O’. | |

The quartile model showed a significant reversion for social stimuli starting from the first quartile (Figure 1A), consistent with Tanaka et al. (2025). In contrast, the quartile-B model showed no significant effect at the first quartile, aligning with the results from the delta and decile models. These findings suggest that the reduction in the conflict effect occurs only within very specific segments of the RT distribution—particularly in the fastest responses (between .05 and .1 proportions). Additionally, the fourth quartile displayed a marginally larger reversion for faces (*p = .0457*), with a significant difference observed in the quartile-B model (*p < .0001*).

The decile model employed ten data points (e.g., Pratte et al., 2021), at probabilities *.05, .15, .25, .35, .45, .55, .65, .75, .85, and .95* (Figure 2).

| 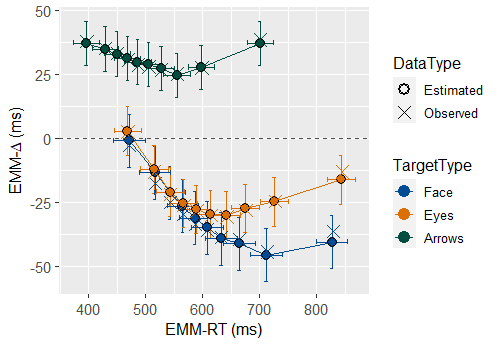 |
| --- |
| **Figure 2.** Delta plots derived from the decile model. The y-axis displays the estimated marginal means of the delta function (EMM-∆), with bars representing the 95% confidence intervals. The x-axis shows the estimated marginal means of reaction time (EMM-RT), with the bars representing the 95% confidence interval for the EMM-RT. Observed values are represented by ‘X’ and estimated values by ‘O’. |

The reversion with social targets in the decile model was significant from the second decile onward. The first decile showed no significant effect, implying that the conflict effect was minimal at this portion of the distribution. Additionally, faces exhibited a larger RCE at deciles nine (*p = .0114*) and ten (*p = .0029*).

# *Trend analyses*

The Trend model, fitted to the delta dataset, included target type, mean reaction time by quantile, and their interaction. It was designed to assess whether the delta function followed a linear or quadratic trend. Random effects accounted for the grouping structure, similarly to the Delta model, with the formula as follows:

*delta ~ TargetType * poly(meanRT, degree = 2) + (1|Study:id) + (1|Study:TargetType) + (1|Study:TargetType:meanRT)*

The results showed stable trends for social targets. In contrast, for non-social stimuli, the findings were sensitive to the trimming procedure and quantile selection.

| **Table 3.** Results of the trend analyses by model and target type | | | | | | |
| --- | --- | --- | --- | --- | --- | --- |
|  | **Linear** | | | **Quadratic** | | |
| **Quantile Trend model** | | | | | | |
| **Target type** | **coeff.** | **t ratio** | **p value** | **coeff.** | **t ratio** | **p value** |
| Face | -.200 [-.23, -.17] | -14.89 | < .0001 | .0003 [.0002, .0005] | 6.92 | < .0001 |
| Eyes | -.092 [-.13, -.06] | -6.37 | < .0001 | .0003 [.0002, .0004] | 6.16 | < .0001 |
| Arrows | -.007 [-.03, .02] | -0.80 | 1.00 | .0003 [.0002, .0004] | 7.04 | < .0001 |
| **Quartile Trend model** | | | | | | |
| Face | -.213 [-.25, -.17] | -13.19 | < .0001 | .0004 [.0002, .0005] | 5.83 | < .0001 |
| Eyes | -.098 [-.14, -.06] | -5.72 | < .0001 | .0003 [.0002, .0005] | 5.48 | < .0001 |
| Arrows | -.048 [-.08, -.02] | -4.32 | < .0001 | .0003 [.0001, .0004] | 4.30 | .0001 |
| **Decile Trend model** | | | | | | |
| Face | -.178 [-.20, -.15] | -17.32 | < .0001 | .0003 [.0002, .0004] | 8.62 | < .0001 |
| Eyes | -.109 [-.14, -.08] | -9.84 | < .0001 | .0003 [.0002, .0004] | 10.17 | < .0001 |
| Arrows | -.015 [-.03, .002] | -2.13 | .1005 | .0003 [.0002, .0003] | 8.23 | < .0001 |
| **Non-trimmed Trend model** | | | | | | |
| Face | -.203 [-.24, -.17] | -14.20 | < .0001 | .0003 [.0002, .0004] | 7.13 | < .0001 |
| Eyes | -.052 [-.09, -.02] | -3.55 | .0012 | .0001 [4.4e-06, .0002] | 2.52 | .0348 |
| Arrows | -.016 [-.04, .01] | -1.60 | .3312 | .0001 [-4.9e-05, .0002] | 1.24 | .6434 |
| ***Note.*** These analyses were performed using the emtrends functions from the emmeans R package (Lenth, 2023) on the Trend models. | | | | | | |

In addition, faces exhibited a significantly more negative linear coefficient than both eyes and arrows across all models assessed. Similarly, eyes showed a more negative slope than arrows in every model except the non-trimmed trend model. in all the models but the non-trimmed trend model. Overall, these results suggest that social stimuli have a more pronounced negative slope than arrows, with faces displaying the steepest negative slope of all.

**REFERENCES**

Barr, D. J. (2013). Random effects structure for testing interactions in linear mixed-effects models. Frontiers in Psychology, 4, Article 328. https://doi.org/10.3389/fpsyg.2013.00328

Castel, A. D., Balota, D. A., Hutchison, K. A., Logan, J. M., & Yap, M. J. (2007). Spatial attention and response control in healthy younger and older adults and individuals with Alzheimer's disease: evidence for disproportionate selection impairments in the Simon task. *Neuropsychology*, *21*(2), 170–182. <https://doi.org/10.1037/0894-4105.21.2.170>

Dalmaso, M., Galfano, G., & Castelli, L. (2023). Are eyes special? Gaze, but not pointing gestures, elicits a reversed congruency effect in a spatial Stroop task. *Atten Percept Psychophys*. <https://doi.org/10.3758/s13414-023-02774-6>

Kuznetsova, A., Brockhoff, P. B., & Christensen, R. H. B. (2017). lmerTest package: Tests in linear mixed effects models. Journal of Statistical Software, 82(13), 1–26. <https://doi.org/10.18637/jss.v082.i13>

Lenth, R. (2023). emmeans: Estimated Marginal Means, aka Least-Squares Means_. R package version 1.9.0. <https://CRAN.R-project.org/package=emmeans>

Meteyard, L., & Davies, R. A. I. (2020). Best practice guidance for linear mixed-effects models in psychological science. Journal of Memory and Language, 112, Article 104092. https://doi.org/10.1016/j.jml.2020.104092

McCullagh, P. and Nelder, J.A. (1989) Generalized Linear Models. 2nd Edition, Chapman and Hall, London.
<http://dx.doi.org/10.1007/978-1-4899-3242-6>

Ponce, R., Lupiáñez, J., González-García, C., Casagrande, M., & Marotta, A. (2024). Exploring the spatial interference effects elicited by social and non-social targets: A conditional accuracy function approach. British Journal of Psychology, 00, 1–20. <https://doi.org/10.1111/bjop.12735>

Pratte M. S. (2021). Eriksen flanker delta plot shapes depend on the stimulus. *Attention, perception & psychophysics*, *83*(2), 685–699. <https://doi.org/10.3758/s13414-020-02166-0>

Schielzeth, H. and Nakagawa, S. (2013) Nested by Design: Model Fitting and Interpretation in a Mixed Model Era. Methods in Ecology and Evolution, 4, 14-24. <https://doi.org/10.1111/j.2041-210x.2012.00251.x>

Tanaka, Y., Oyama, T., Ishikawa, K., & Okubo, M. (2025). Temporal dynamics of activation and suppression in a spatial Stroop task: A distribution analysis on gaze and arrow targets. Attention, perception & psychophysics, 87(2), 367–383. https://doi.org/10.3758/s13414-024-02993-5
